# Effect of the Sulfonation on the Swollen State Morphology of Styrenic Cross-Linked Polymers

**DOI:** 10.3390/polym12030600

**Published:** 2020-03-06

**Authors:** Chiara Dalla Valle, Marco Zecca, Federico Rastrelli, Cristina Tubaro, Paolo Centomo

**Affiliations:** Department of Chemical Sciences, University of Padova, Via Marzolo 1, 35131 Padova, Italy

**Keywords:** swollen state porosity, crosslinked polymers, sulfone bridges, ISEC, CP-MAS ^13^C-NMR

## Abstract

The chemical structure and morphology of a set of sulfonic gel-type poly(styrene-divinylbenzene) resins (2 mol% DVB) prepared with different synthetic approaches were investigated by solid state NMR, Inverse Size Exclusion Chromatography (ISEC), FT-IR and elemental analysis to compare their swollen state structure. FT-IR and solid state NMR clearly show that the sulfonation mainly occurs in the para- position with respect the main polymer chain. Sensible proportions of sulfone bridges were found in the materials obtained with oleum and chlorosulfonic acid. With oleum, the presence of the sulfone bridges is clearly associated to a reduced ability to swell in the water medium relative to the proton exchange capacity. This highlights the cross-linking action of the sulfone bridges according to ISEC results, showing a high proportion of a dense polymer fraction in the swollen material. An even higher degree of sulfone-bridging, lower swelling ability, and a high proportion of a dense polymer fraction in the swollen material are found in the resin obtained with chlorosulfonic acid. As a matter of fact, Cross Polarization Magic Angle Spinning Nuclear Magnetic Resonance (CP-MAS ^13^C-NMR), elemental analysis, and ion exchange capacity, show that oleum and chlorosulfonic acid produced resins with remarkably smaller pores and lower swollen gel volume in polar solvents, with respect to concentrated sulfuric acid.

## 1. Introduction

Sulfonic resins based on polystyrene-divinylbenzene (PS-DVB) are widely used in the fields of acid catalysis, ion exchange, and separation processes [1,2]. They must be used in the presence of a suitable liquid phase (water, an aqueous solution, or a polar organic solvent) able to swell the polymer framework: under these conditions an extensive nanometer size porosity with a surface area of hundreds of square meters per gram is generated [3,4,5]. The investigation of the morphological features of this porous system, representing the actual working arena of the resin requires suitable solution state techniques, such as Inverse Size Exclusion Chromatography (ISEC) [3,5,6,7], Electron Paramagnetic Resonance (EPR) investigation of the rotational motion of suitable nitroxide probes [8,9], and time domain NMR spectroscopy [10], to rationalize the physico-chemical behaviour of these materials [11,12,13].

The direct sulfonation of PS-DVB resins with either sulfuric acid or oleum, or with chlorosulfonic acid and subsequent alkaline hydrolysis [14], introduces not only the sulfonic groups, but also sulfone bridges between pairs of phenyl rings. These bridges not only reduce the effective degree of sulfonation, but also represent a form of cross-linking which adventitiously adds to the cross-links produced by DVB. The elucidation of post cross-linking of PS-DVB resins at the molecular level has been addressed so far only in an early report by Goldstein and co-workers [15] and in a more recent one by Sherrington [16]. However, they were both devoted to the formation of cross-links upon Friedel–Crafts reactions catalyzed by Lewis acids such as FeCl_3_ and ZnCl_2_. In one case [16], this was the unpredicted result of the chloromethylation of PS-DVB with (chloromethyl)methylether during the production of anion-exchange resins. Solid state ^13^C-NMR allowed to characterize the parent resin, all the intermediate products and the final ion-exchanger. The degree of post cross-linking (methylene bridges between phenyl ring pairs) was quantified in 50–60%. As solid state ^13^C-NMR still represents a state-of-the-art technique for the characterization of polymers [17] in the solid state, we decided to take a similar approach to address the problem of sulfone bridges formation during PS-DVB sulfonation with different agents. In this way, a semi-quantitative evaluation of the content of sulfone bridges was achieved. Moreover, it is well known that changes in the cross-linking degree of resins affects their swelling behaviour (as a rule of thumb, the higher the former, the smaller the latter). Therefore, we also investigated on the effect of the additional sulfone-bridge cross-links on the swollen state morphology of the ion-exchangers. For this purpose, the swollen sulfonated resins were characterized by ISEC in order to disclose differences in the porosity of ion-exchangers prepared with different sulfonating agents. All these pieces of information might be useful for better controlling the production of sulfonated ion-exchange resins and tailoring their morphology to specific practical applications under solid-liquid condition processes.

## 2. Materials and Methods

The starting material for the sulfonation is a gel-type styrene-co-divinylbenzene polymer with a nominal DVB content of 2% mol which was provided by Spolchemie, Usti nad Labem, Czech Republic. Reagents and solvents were purchased from Sigma-Aldrich and used as received.

### 2.1. Sulfonation with Concentrated Sulfuric Acid

Ca. 2 g of polymer were allowed to swell overnight in the least volume of 1,2-dichloroethane (DCE) as the solvent (10–20 mL), in a jacketed glass reactor equipped with a back condenser. 50–100 mL of 98% H_2_SO_4_ (acid:solvent, 5:1 *v*/*v*) were slowly added to the swollen polymer. The mixture was heated up to 80 °C for 3 h, under moderate magnetic stirring and then cooled to room temperature. The reaction mixture was diluted with five portions of ca. 30 mL of sulfuric acid with a progressively lower concentration (10 M; 5 M; 2.5 M; 1 M; 0.1 M); a flow of cold water through the jacket of the reactor was used to prevent overheating. Finally, the resin was recovered by filtration, washed with distilled water up to neutral pH and dried at 110 °C.

### 2.2. Sulfonation with Oleum

The procedure is the same used for the sulfonation with sulfuric acid, but oleum replaced sulfuric acid as the sulfonating agent.

### 2.3. Sulfonation with Chlorosulfonic Acid

The resin (ca. 2 g) was pre-swollen overnight with 1,2-dichloroethane in a glass jacketed reactor. 24 mL of 1,2-dichloroethane and 16 mL of HClSO_3_ were added under magnetic stirring and the mixture was let to react for three hours at 42 °C. Subsequently, 50 mL of cold distilled water was added dropwise to the reaction mixture, maintained at low temperature with an ice bath. The chlorosulfonic resin was then recovered by vacuum filtration (G3 gooch), washed with distilled water until neutral pH and suspended in 40 mL of a 2% wt solution of NaOH in 1,4-dioxane:water 1:1 *v*/*v*, at 40 °C for two hours. The resin was again recovered by vacuum filtration (gooch G3) and washed with a 10% HCl solution in 1,4-dioxane:water 1:1 *v*/*v*, up to the complete removal of the chloride ions from the filtrate (AgNO_3_ test). The solid was finally washed with 50 mL methanol and dried at 110 °C overnight.

### 2.4. Determination of the Proton-Exchange Capacity of Sulfonic Resins

Proton exchange capacity (PEC) is determined by acid-base back titration. About 100 mg of resin was exactly weighted after drying overnight at 110 °C in an oven. Each sample was treated with 10.0 mL of a standard aqueous solution (ca. 0.1 M) of NaOH in a 50 cm^3^ stoppered Erlenmeyer flask. The suspension was mechanically swirled (orbiting plate) overnight. To take into account the possible reaction with atmospheric CO_2_, a blank with 10.0 mL of the NaOH solution was prepared as well. The solutions (sample and blank) were eventually titrated with standard 0.1 M HCl and phenolphthalein as the indicator. The apparent specific content of sulfonic groups was calculated as the difference of the initial and final millimoles of NaOH (corrected for over-consumption due to CO_2_, if any) divided by the resin mass.

### 2.5. ISEC Investigation

The resin was loaded as the stationary phase in a void HPLC column and the elution volumes of standard solutes with known molecular size are measured in a HPLC apparatus modified ad-hoc [6]. The measurements were performed using a 0.2 M sodium sulfate solution as the mobile phase and D_2_O, sugars and dextrans as standard solutes. Further details can be found in the Supplementary Information and, for an extensive description of the technique, in ref. [3].

### 2.6. Solid State NMR

NMR spectra in the solid state were collected on a Varian 400 equipped with a narrow bore, triple resonance T3 Magic Angle Spin (MAS) probe spinning 4 mm rotors and operating at ^1^H and ^13^C frequencies of 400.36 and 100.68 MHz, respectively. The nominal temperature of the probe was always set to 298 K. Cross Polarization Magic Angle Spinning Nuclear Magnetic Resonance (CP-MAS ^13^C NMR) spectra were acquired at 10 kHz MAS with 1200 scans and a repetition delay of 3 s. The contact time for CP was 1 ms, and an acquisition time of 50 ms was used. The chemical shifts were referenced against the CH_2_ resonance observed for adamantane at 38.48 ppm with respect to the signal for neat tetramethylsilane (TMS). In order to homogenize the samples, resin beads were ground to obtain a fine powder prior to insertion into the rotor.

## 3. Results and Discussion

### 3.1. Proton Exchange Capacity (PEC)

The comparison of the PECs of the ion-exchange resins obtained with different sulfonation methods with the respective sulfur contents from the elemental analysis can give valuable information. The PECs of the polymer materials determined by back-titration are reported in Table 1.

The PEC of a gel-type styrenic resin cannot exceed 5.4 mmol H^+^ g^−1^, corresponding to the functionalization of all the aromatic rings with one sulfonic group each. The simple inspection of PECs suggests that oleum gave an oversulfonated resin (*Gel_oleum*), and concentrated H_2_SO_4_ introduced more or less one sulfonic group per phenyl ring in *Gel_H_2_SO_4_*.

This simple analysis would also suggest that sulfonation was not quantitative in *Gel_HClSO_3_*, which seems rather surprising. Whereas sulfuric acid and oleum are neither soluble in the swelling agent (DCE) nor able to swell the starting resin, chlorosulfonic acid is miscible with DCE. On this basis, chlorosulfonic acid should access the swollen polymer framework easily in comparison with sulfuric acid or oleum. The difficulty of the latter two sulfonation agents to diffuse into the polymer framework is shown by the egg-shell distribution of sulfonic groups in partially sulfonic resins [11]. In these cases, the sulfonation starts from the external surface, then the first sulfonic (and hydrophilic) layer is swollen by H_2_SO_4_ (or oleum) which sulfonates a second layer just beneath the surface and so on. However, the comparison of PECs with the total sulfur and carbon from the elemental analysis gives a more detailed and appropriate insight on the effects of the different sulfonation methods.

Sulfur can be distributed among three different types of “sulfo”-groups: sulfonic (−SO_2_OH), sulfone bridges (−SO_2_−) and chlorosulfonic (−SO_2_Cl). The latter were found, in little amount, only in *Gel_HClSO_3_* as shown by the presence of chlorine in the material. To estimate the actual degree of sulfonation of the phenyl rings we assumed that the resins were essentially sulfonic polystyrene with a formula weight C_2_H_3_-C_6_H_5_·*x*SO_3_ (we neglected both the presence of DVB, which is nominally only 2% of the resin and the different nature of the sulfo-groups), where *x* is straightforward to be obtained from the ratio of the C and S weight percentages (Equation (1)):

(1) $$x=8\times \frac{S\left(\%wt\right)\times AW\left(C\right)}{AW\left(S\right)\times C\left(\%wt\right)}\approx 3\times \frac{S\left(\%wt\right)}{C\left(\%wt\right)}$$

This number is also the degree of sulfonation, defined as the average number of “sulfo” groups per phenyl ring. The values of *x* show that the starting gel-type resin is fully (at least) sulfonated in each case and confirm that a remarkable oversulfonation takes place with oleum. In line with previous reports of sulfonation of PS-DVB resins with oleum exceeding the limit of one sulfonic group per each phenyl ring of the polymer [18], the PEC of *Gel_oleum* is consistent with the presence of 1.17(5) sulfonic groups per phenyl ring i.e., ca. 17% of the phenyl rings bear two acidic groups. However, this does not entirely account for the total content of sulfur of *Gel_oleum*. It can be estimated that there are on average 0.15(5) sulfone bridges per phenyl ring, which amounts to ca. 11% of total sulfur in the resin. This also implies that ca. 30% of the phenyl rings are involved in the formation of bridged pairs and if we assume that there are no triply sulfonated rings, almost half of the phenyl rings of *Gel_oleum* are doubly sulfonated in one form or the other.

The analytical data of Table 1 support an even more extensive formation of sulfone bridges in *Gel_HClSO_3_*. In this case oversulfonation does not take place and this mainly goes to detriment of the PEC, with ca. 77% of the phenyl rings bearing only one sulfonic group. As mentioned above, the presence of chlorosulfonic groups also gives a contribution to the difference between the total sulfur amount of *Gel_HClSO_3_* and its PEC. However, it is relatively little (0.11 mmol·g^−1^ out of 5.40 mmol·g^−1^ of total sulfur) and only ca. 2% of the phenyl rings bear -SO_2_Cl groups. The remaining ca. 21% of total sulfur of *Gel_HClSO_3_* must be accounted for by the formation of sulfone bridges, which implies that more than 40% of them are involved in the formation of bridged pairs, but only about one fifth are doubly sulfonated. HClSO_3_ is therefore more apt to form sulfone bridges than oleum, which gives only 11% of total sulfur in this form, although it proves to be a weaker sulfonating agent in the conditions employed herein.

Finally, the analytical data of *Gel_H_2_SO_4_* show that neither the oversulfonation nor the formation of sulfone bridges occur with sulfuric acid. Under the conditions employed in this work, the sulfonation leads readily to an essentially fully sulfonic resin.

All these results are completely in line with the well-known circumstance that the formation of sulfone bridges is a side reaction of the sulfonation with oleum and chlorosulfonic acid [11,15,19,20]. According to the literature [21], it involves the acid catalyzed condensation of two sulfonic group, in which the use of a very strong acid and dehydrating agent such as oleum is expected to favour both the protonation of the sulfonic groups and the subsequent elimination of water. Moreover, in doubly sulfonated rings, the acidity of the sulfonic groups is significantly enhanced [18], due to the deactivation of the aromatic unit, and the protonation step is expected to be even more effective.

### 3.2. Morphological Characterization in the Swollen State

The swollen state morphology of cross-linked polymers can be conveniently investigated by means of ISEC [7,22,23]. This kind of materials can have specific surface areas and pore volumes much larger in the swollen state than in the dry state. Gel-type resins, for instance, are essentially non-porous and have specific surface areas of a few square-meters per gram only when dry. However, they can swell extensively in proper liquids. The intrusion of the molecules of the swelling agent into the polymer framework separates the polymer chains from one another. The framework expands and the space between the separated chains is filled by the molecules of the swelling agent. This process produces a gel in which the cavities, created by the separation of the chains, have characteristic size comparable to those of micropores and small mesopores. The specific gel volume and surface area can amount to a few cubic centimetres per gram and several hundreds of square meters per gram, respectively. Under these conditions, the interior of the polymer framework can be accessed from the liquid phase in contact with it and exploited for chemical purposes.

In this case, the resin morphology is best described with the Ogston model, which depicts the polymer chains as randomly oriented rigid cylindrical rods, with high aspect ratios, and the pores as void spaces among them [24]. In this model the characteristic quantity is the polymer chain concentration, the sum of the lengths of the rods which are contained in a unit volume of gel and which is generally expressed in nm·nm^−3^. Real swollen materials are generally described as a number of discrete gel fractions, each with its volume and characteristic polymer chain concentration. The result of ISEC analysis based on the Ogston model is therefore a volume distribution of different values of polymer chain concentration. As an alternative the cylindrical pore model could also be used, where the pores are depicted as cylinders with a specific pore diameter. This provides a much more direct estimation of the sizes of the cavities in the expanded polymer framework, although it is clear that nothing similar to pores in rigid materials can hardly exist in this case and that the Ogston model is closer to the real situation. The general relationship between the two models is that higher polymer chain concentrations correspond to smaller “pore diameters” and vice versa.

**Figure 1:**
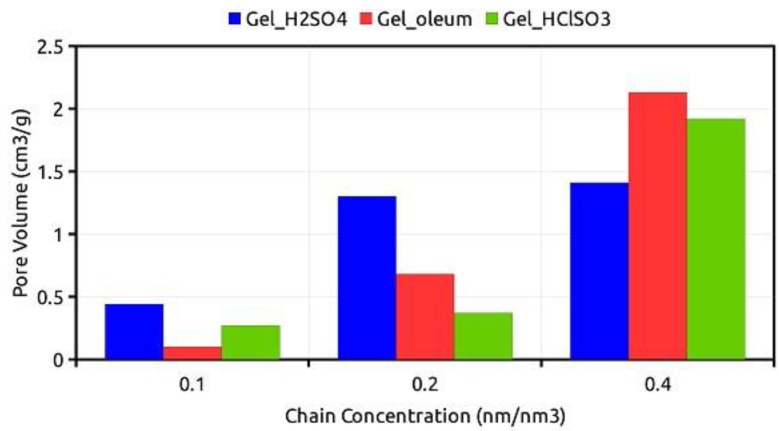
Distribution of the polymer chain concentration of *Gel_H_2_SO_4_*, *Gel_oleum* and *Gel_HClSO_3_*, according to ISEC.

The ISEC characterization of *Gel_H_2_SO_4_*, *Gel_oleum* and *Gel_HClSO_3_* provides important information on the effects of the sulfonation method on the nanometer scale morphology of the swollen gels. They are presented herein using the Ogston model, hence each material is divided in a set of six discrete domains with polymer chain concentrations ranging from 0.1 to 2 nm nm^−3^, which approximately correspond to pore diameters from 6 to 1 nm. The results of ISEC characterization are summarized in Table 2 and illustrated in Figure 1.

Due to the hydrophilic nature of the sulfonic PS-DVB resins, an aqueous solution of sodium sulfate (0.2 M) was used as the mobile phase in the ISEC runs. The presence of the electrolyte is necessary to suppress enthalpic contributions to the partition of the standard solutes between the stationary and mobile phases. The polymer chain concentration exceeds 0.4 nm·nm^−3^ and most of the polymer length (hence mass) is always found in the 0.4 nm·nm^−3^ and 0.2 nm·nm^−3^ fractions. However, whereas in *Gel_H_2_SO_4_* these fractions have approximately the same volume (ca. 45% of the total gel volume), in *Gel_oleum* and *Gel_HClSO_3_* the 0.4 nm·nm^−3^ has a much higher relative volume (ca. 75% of the total gel volume in both cases). This shows that *Gel_oleum* and *Gel_HClSO_3_* have a lower swelling ability in water than *Gel_H_2_SO_4_*. Moreover, looking at the total gel volume *Gel_oleum* seems to swell slightly better than *Gel_HClSO_3_*. The relatively little swell-ability of *Gel_HClSO_3_* could be simply attributed to its lower PEC (4.16 mmol·g^−1^), which makes it slightly less hydrophilic than *Gel_oleum* and *Gel_H_2_SO_4_* (PECs = 5.42 and 5.20 mmol·g^−1^, respectively). However, this argument cannot hold in the comparison of *Gel_oleum* and *Gel_H_2_SO_4_*. In this case, the reduced ability of *Gel_oleum* to swell could depend on the formation of sulfone bridges during the sulfonation. In fact, *inter*-chain bridges represent an additional form of cross-linking and the sulfur balance discussed in the previous section shows that this could amount up to 15%. Such an additional cross-linking does not seem compatible with the relatively small difference in the swelling ability between *Gel_oleum* and *Gel_H_2_SO_4_* and this suggests that most of the sulfone bridges could be of an *intra*-chain nature. Nevertheless, ISEC provides evidence that the formation of sulfone bridges is associated to the decrease in the swelling ability of sulfonic PS-DVB resins. The number of sulfone bridges estimated from the sulfur balance of *Gel_HClSO_3_* in even higher and we cannot rule out that this could contribute to its relatively low swelling ability in addition to its lower PEC and hydrophilicity.

### 3.3. ATR-FT-IR Characterization

**Figure 2:**
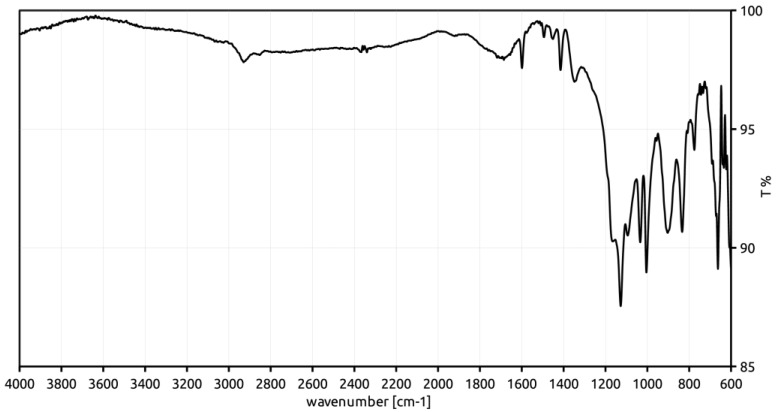
FT-IR spectrum of *Gel*_*H_2_SO_4_*.

**Figure 3:**
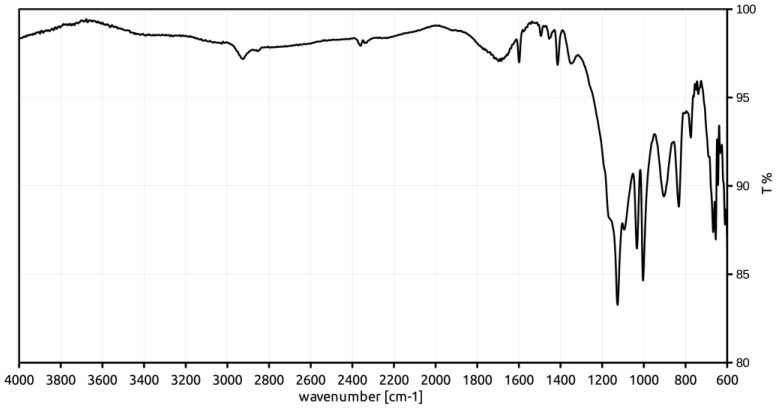
FT-IR spectrum of *Gel_HClSO_3_*.

**Figure 4:**
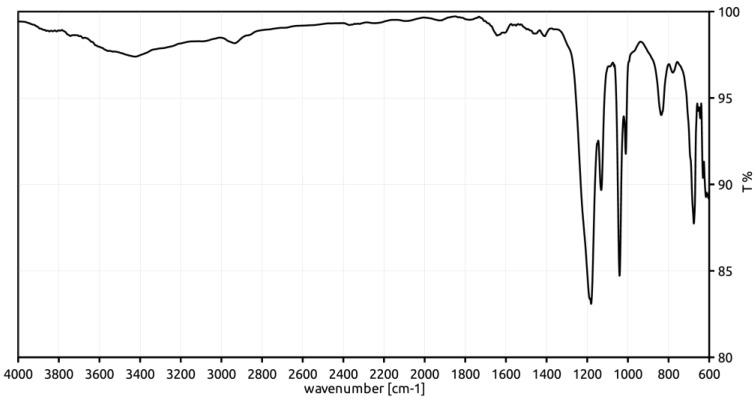
FT-IR spectrum of *Gel_oleum*.

To support the relationship between the observed increased crosslinking degree and the presence of sulfone bridges, the sulfonic materials have been characterized with FT-IR spectroscopy to identify the characteristic signals of the expected functional groups. The spectra, collected in attenuated total reflectance (ATR) geometry, are reported in Figure 2, Figure 3 and Figure 4.

The relevant FT-IR data and the assignments of signals to vibrational modes are summarized in Table 3 [25].

All the spectra contain peaks which can be attributed to the sulfonic groups, such as those at 1348 cm^−1^ and 1033 cm^−1^. The signals at 836 and 772 cm^−1^ suggest that the functionalization of the phenyl rings takes place mainly in the ortho- and para-positions with respect to the alkyl chains of the polymer framework. However, para- position appears the most likely, due to its relatively high distance from the sterically bulky alkyl chains of the polymer framework. Also, the signal at 907 cm^−1^ can be reasonably assigned to the stretching vibration of the S-O bond of the −SO_3_H group, in spite of a significant shift from the literature (897 cm^−1^).

The signal at 1166 cm^−1^ (present as a shoulder in the spectra of *Gel_oleum* and *Gel_HClSO_3_* materials) has been considered by some authors as diagnostic for the sulfone bridges. In fact, the diphenyl sulfones show the symmetric stretching vibration of two S-O bonds between 1160 and 1164 cm^−1^ [26,27]. Conversely, a signal at 1172 cm^−1^, due to the symmetric stretching vibration of the S=O bonds of the SO_3_H group, is also reported for sulfonic polystyrene materials [25]. Therefore, the signal at 1166 cm^−1^ cannot unambiguously support the presence of sulfone bridge in the sulfonic resins.

Moreover, the SO_2_-antisymmetric stretching of the −SO_2_Cl group is not recognized in the FT-IR characterization of *Gel_HClSO_3_*. Accordingly, the chlorine content is very low (0.11 mmol g^−1^) and the conversion of the sulfonyl chloride units into sulfonic groups in the second stage of sulfonation process can be considered almost complete.

To summarize, the FT-IR characterization of the sulfonic materials is compatible with the possible formation of sulfone bridges and suggest the para- position of the styrenic rings as the most probable position for the sulfonation.

### 3.4. Solid State CP-MAS ^13^C-NMR Characterization

**Figure 5:**
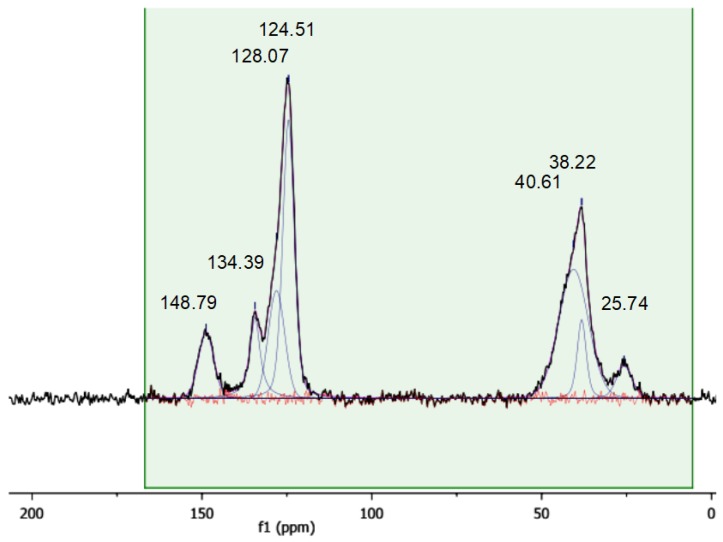
^1^H-^13^C CP MAS spectrum of *Gel_H_2_SO_4_*. Contact time 1 ms, MAS rate 10 kHz, T = 25 °C.

**Figure 6:**
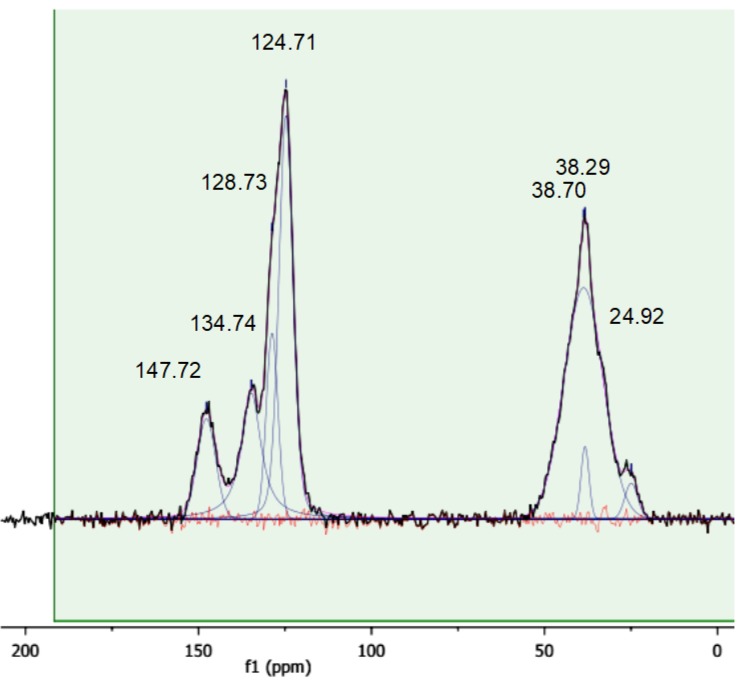
^1^H-^13^C MAS spectrum of *Gel_oleum*. Contact time 1 ms, MAS rate 10 kHz, T = 25 °C.

**Figure 7:**
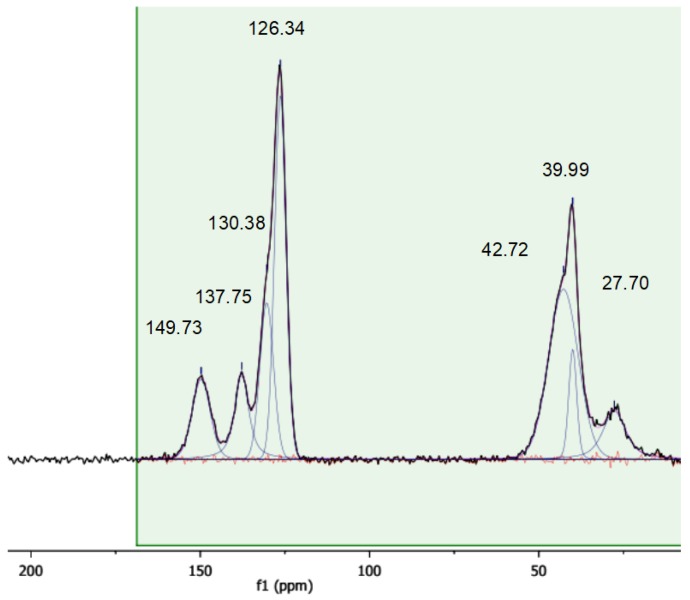
^1^H ^13^C MAS spectrum of *Gel_HClSO_3_*. Contact time 1 ms, MAS rate 10 kHz, T = 25 °C.

**Figure 8:**
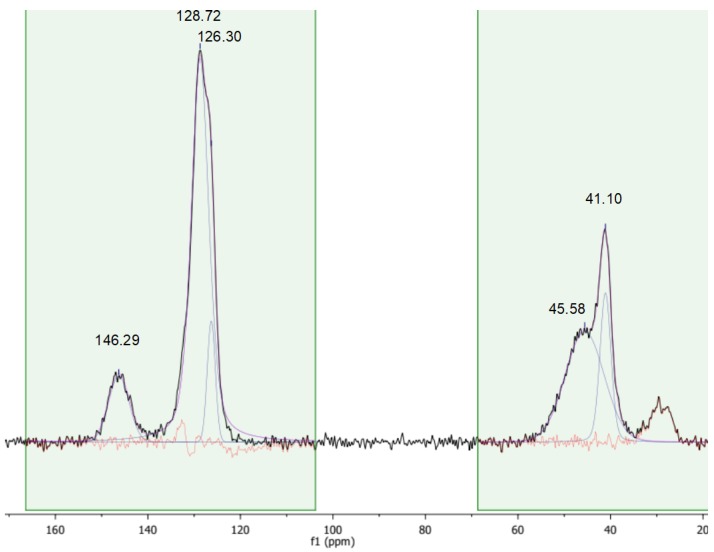
^1^H ^13^C MAS spectrum of the starting gel-type poly(styrene-divinylbenzene) (2% mol DVB). Contact time 1 ms, MAS rate = 10 kHz, T = 25 °C.

A more detailed insight into the chemical nature of the sulfonic resins can be obtained with solid state CP-MAS NMR (Figure 5, Figure 6 and Figure 7). For the sake of comparison, the NMR spectrum of the starting cross-linked polymer has also been collected (Figure 8). The occurrence of isotropic chemical shifts vs. spinning sidebands has been checked for, by comparing spectra registered at different MAS speeds. Deconvolution of the observed signals has been carried out taking into account the chemical non-equivalence of the different carbon atoms.

The signals at 41.10 ppm and 45.58 ppm in the spectrum of the starting material (Figure 8) can be assigned to the backbone carbons. The signal at 146.29 ppm is due to the quaternary carbon of the aromatic rings, whereas the signals at 128.72 and 126.30 ppm have been attributed to non-functionalized aromatic carbons [16].

The signals of sulfonic resins, summarized in Table 4, have been assigned according to the literature [28,29,30]. The positions of the carbon atoms of the aromatic rings given hereafter will be referred to the position of the polymeric chain.

The spectra of alkylbenzenes or 1,3-diphenylpropane and of p-toluensulfonic acid in CDCl_3_ [31] can be used to attribute the signals in the aromatic region by comparison. Accordingly, the aromatic carbon atom directly attached to the polymeric chain (ipso) resonates in the 146–150 ppm in all the materials. In the parent gel resin (non-sulfonic) the resonances of the ortho and meta carbon are superimposed at 128.7 ppm (128.39 and 128.26 ppm in 1,3-diphenylpropane) and the signal at 126.3 ppm is assigned to the para- carbon atom. A signal in the 124.5–126.3 ppm range is found also in the sulfonic polymers, but in this case, it can be attributed to the meta carbon atom, by analogy with p-toluensulfonic acid (125.45 ppm). The para position is the preferred one for the sulfonation of polystyrene [28,29,30,32] and the carbon atom bearing the sulfonic group resonates in the 134.4–137.8 ppm range. Not only is this signal absent in the spectrum of the parent gel resin, but its position is also consistent with the solution-state spectrum (CDCl_3_) of p-toluensulfonic acid, where the sulfonated carbon atoms resonate at 138.49 ppm. Hence, in view of the degree of sulfonation obtained from the mass balance of sulfur, no unsubstituted para carbon atoms should be present hence.

**Figure 9:**
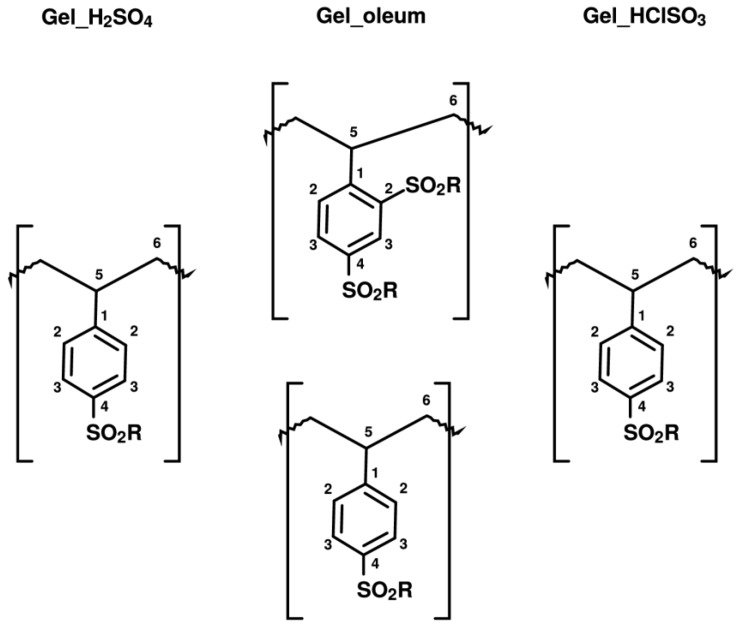
Chemical structures of the samples, expected on the basis of comparison between elemental analysis and ion exchange capacities. The aromatic carbon atoms with similar chemical shifts are labeled with the same letters. The R group is either the −OH moiety (in the case of sulfonic group) or an aromatic ring (in the case of sulfone bridge).

As shown above in *Gel_oleum,* ca. 33% of the aromatic rings are doubly sulfonated, but its spectral pattern is practically the same of the other two sulfonic resins. We therefore speculate that the sulfonated carbon atoms resonate at the same frequency irrespective of their position or that their resonances are so close that they cannot be resolved. The second sulfonation of the aromatic rings of *Gel_oleum* most likely occurs in the ortho- position, which is favoured by the orienting effects of both the (alkyl) polymeric chain and of the first “sulfo” group, assuming that the para position is sulfonated first. This is consistent with the well-know regiochemistry of aromatic electrophilic substitutions. In fact, the polymer chains can be considered as alkyl substituents of the phenyl rings at the ipso carbon atoms. Alkyls are electron-donating, para- and ortho-directing groups. As the polymeric chains are very bulky, the para- position should be preferred for the first sulfonation. In addition, the first sulfonic group introduced in the phenyl ring, which is electron withdrawing, is meta- directing (with respect to its own position). Thus, it should also contribute to the sulfonation of the ortho- position with respect to the ipso carbon atom. This is qualitatively confirmed by the analysis of the peak intensity in the spectra of the sulfonic resins (Table 5; for sake of clarity the carbon atoms are also numbered according to the structural scheme represented in Figure 9).

Although the intensity of cross-polarized signals cannot be directly related to the number of resonating spins, the ratio between the areas of selected signals can be compared for similar samples. For *Gel_H_2_SO_4_* and *Gel_HClSO_3_*, which have no doubly-sulfonated aromatic rings, the intensity ratios ortho/sulfonated and meta/sulfonated are similar. By contrast, for *Gel_oleum* the intensity ratios ortho/sulfonated and meta/sulfonated are much lower and much higher, respectively. This suggests, at least qualitatively, that the number of carbon atoms in ortho to the polymeric chain is smaller in *Gel_oleum* and supports the hypothesis of the second sulfonation of its aromatic rings in this position. A complete sulfonation in the ortho- position is anyway ruled out by the NMR data and also by the presence of the signal at 836 cm^−1^ in the IR spectra of the sulfonic resins.

## 4. Conclusions

The sulfonation of a 2% cross-linked, gel-type polystyrene-divinylbenzene resin with concentrated sulfuric acid, oleum and chlorosulfonic acid produces ion-exchange resins (*Gel_oleum*, *Gel_H_2_SO_4_*, *Gel_HClSO_3_*) with different properties. The conditions employed allow to achieve full sulfonation of the resin and, with oleum even oversulfonation. Hence *Gel_oleum* has a PEC slightly higher than the ceiling value for a fully mono-sulfonic resin and, of course, higher than the PECs of *Gel_H_2_SO_4_* and *Gel_HClSO_3_*. In view of its relatively high PEC, *Gel_oleum* is expected to be the most hydrophilic material hence to have the largest swelling ability in aqueous environment. In spite of this its swollen gel volume, as measured by ISEC, is somewhat lower than the swollen gel volume of *Gel_H_2_SO_4_*. The detailed morphological ISEC analysis also shows that in *Gel_oleum* ca. 73% of the gel volume is represented by the relatively most dense fraction (0.4 nm·nm^−3^), while it is only 44% in *Gel_H_2_SO_4_*. This counter-intuitive result can be attributed to the presence of sulfone bridges in *Gel_oleum*. From the mass balance of sulfur, it can be estimated that they connect ca. 30% of the aromatic rings of *Gel_oleum* while there are none in *Gel_H_2_SO_4_*. This implies that the actual cross-linking degree of *Gel_oleum* is higher (and hence its swelling ability is lower) than that of *Gel_H_2_SO_4_*. These conclusions are also supported by CP-MAS ^13^C-NMR spectroscopy. *Gel_HClSO_3_* has the lowest swelling ability in aqueous environment and the highest proportion of sulfone bridges relative to both the number of aromatic rings and the total sulfur amount. However, it also has the lowest PEC (hence hydrophilicity), which is mostly the consequence of the aptitude of HClSO_3_ to give a high proportion of sulfone bridges and its inability to give oversulfonation (the contribution of the incomplete hydrolysis of the intermediate −SO_2_Cl groups to −SO_3_H is almost negligible). The reduced swelling ability of *Gel_HClSO_3_* could therefore arise from one or both these causes. Further investigation is needed to find which one plays the most important role.

## Figures and Tables

**Table 1:** Analytical data on the composition of the sulfonic resins.

Sample ^1^	PEC ^2^	C% wt	Total S% wt	Cl% wt	x ^3^
*Gel_oleum*	5.51	44.96	19.99 (6.24) ^2^	−	1.33
*Gel_H_2_SO_4_*	5.20	50.64	17.31 (5.40) ^2^	−	1.02
*Gel_HClSO_3_*	4.16	50.40	17.33 (5.40) ^2^	3.9 ^4^ (0.11)	1.03

^1^ approximate formula weight = C_2_H_3_-C_6_H_5_·xSO_3_; ^2^ mmol·g^−1^; ^3^ average number of sulfonic groups *per* phenyl ring; ^4^ due to residual SO_2_Cl from incomplete basic hydrolysis.

**Table 2:** ISEC characterization of *Gel_H_2_SO_4_*, *Gel_oleum*, *Gel_HClSO_3_*.

Polymer Chain Concentration (nm nm^−3^)	Polymer Chain Concentration (nm nm^−3^)		
	*Gel_H_2_SO_4_*	*Gel_oleum*	*Gel_HClSO_3_*
0.1	0.44	0.10	0.27
0.2	1.30	0.68	0.37
0.4	1.41	2.13	1.92
0.8	0.00	0.00	0.00
1.5	0.00	0.00	0.00
2.0	0.00	0.00	0.00
**Total gel volume (mL g^−1^)**	**3.15**	**2.91**	**2.56**

**Table 3:** Diagnostic signals of sulfonic poly(styrene-divinylbenzene) materials, with the pertinent assignments.

Peak/Band (cm^−^^1^)	Assignment
1599, 1495, 1413	Skeleton stretching vibrations of the benzene ring
1451	scissor vibration of CH_2_ group
1348	antisymmetric stretching vibration of the S=O bonds
1033	symmetric stretching vibration of the −SO_3_^−^ ion
1126, 1097	in-plane skeleton vibration of the benzene ring. Bands characteristic of disubstituted benzene rings
1004	in-plane bending vibrations of the CH groups for benzene ring
836/831	out-of-plane vibration of the pairs of CH groups in the p-substituted benzene ring (in comparison with p-toluensulfonic acid, 817 cm^−1^)
772	out-of-plane vibration of four CH groups in the o-substituted benzene ring (as the term of comparison, 765 cm^−1^ for o-toluensulfonic acid)

**Table 4:** Signal attribution of ^1^H ^13^C MAS spectra of the sulfonic samples (δ, ppm).

	C _ipso_ (1)	C _para_ (4)	C _ortho_ (2)	C _meta_ (3)	C _aliphatic_	
Parent Gel resin	146.3	126.3	128.7	128.7	45.6	41.1
*Gel_oleum*	147.7	134.7	128.7	124.7	38.7	38.3
*Gel_H_2_SO_4_*	148.8	134.4	128.1	124.5	40.6	38.2
*Gel_HClSO_3_*	149.7	137.8	130.4	126.3	42.7	40.0

**Table 5:** Integrated intensities (absolute units) of the signals of aromatic carbon atoms.

	*Gel_H_2_SO_4_*	*Gel_oleum*	*Gel_HClSO_3_*
A_ipso_ (1)	723.785	1476.143	3467.159
A_sulfonated_ (2)	834.452	2831.201	3574.537
A_ortho_ (3)	1229.298	1716.835	4811.788
A_meta_ (4)	2542.258	5038.13	9517.451
A_sulfonated_/A_ortho_ (4/1)	0.68	1.66	0.75
A_meta_/A_ortho_ (3/1)	2.1	2.87	1.99

## Supplementary Materials

The following are available online at https://www.mdpi.com/2073-4360/12/3/600/s1.
