# Supplementary material for: Effect of the Sulfonation on the Swollen State Morphology of Styrenic Cross-Linked Polymers
Source: Polymers (Basel). 2020 Mar 6;12(3):600. doi: 10.3390/polym12030600 (PMC7182915; doi:10.3390/polym12030600)
Supplement: Supplementary file 1 [file polymers-12-00600-s001.pdf]

ISEC Characterization

In Inverse Size Exclusion Chromatography (ISEC, also referred to as inverse GPC and chromatographic porosimetry) standard eluates of known size are used for the investigation of the porosity of a material, packed inside a chromatographic column [1–5]. It is a chromatographic method, hence non-destructive, based on a standard LC setup. With respect to gas adsorption and mercury intrusion, this technique makes possible to investigate the materials in the presence of a solvent. For polymers swelling can take and make their morphology remarkably different from those at the dry state. To this regard, ISEC appears a unique tool for the assessment of the morphology of swollen polymers (their working state under solid-liquid conditions).

In SEC, different eluates have different elution volumes depending on their steric interaction with the stationary phase (provided enthalpic interaction are negligible or comparable): the bulkier the molecules the smaller their elution volume. Totally excluded molecules are eluted at the column void volume (V_0); molecules small enough to fully permeate the pore volume (V_p) are eluted at (almost) the total column volume V_t (=V_0+V_p). For intermediate-sized molecules the elution volume (V_e) is between these two extremes, depending on their equilibrium partition constant (K, Eq. 1):

$$V_e = V_0 + K \cdot V_p \qquad (1)$$

In the case of purely sterically-driven partition of the eluates, after assuming a model of the pore geometry (e.g. cylindrical pore model or Ogston model) the values of K can be calculated from the pore size and the respective size of the eluates.

If the pores are assumed to be regular cylinders and the solute molecules to be spherical, the partition coefficient K_c is simply:

$$K_c = \left[1 - \left(\frac{d_s}{d_p}\right)\right]^2 \qquad (2)$$

where d_p and d_s are the pore and solute molecule diameters, respectively. This simple model provides a friendly description of the morphology of the swollen gel, but not fully reliable for the assessment of extensive quantities (in particular V_p) [5,6]. A better model of the actual physical state of the swollen gel was developed by Ogston [7], describing the polymer network as an array of randomly oriented rigid rods (with very long length to diameter ratio): as the consequence, the pore system consists in the void spaces among the rods. Under these assumption, the partition coefficient, K_O, can be determined according Eq. 3:

$$K_o = \exp\left[\frac{-\pi \cdot C}{4}\left(d_s + d_c\right)^2\right] \qquad (3)$$

where d_s and d_c are the diameters of the rigid spherical solute and the rigid rods representing the polymer chains (0.4 nm for styrenic polymers), respectively; C is the polymer chain concentration,

representing the total lengths of each rod (or rod section) per volume unit, usually expressed in $nm \cdot nm^3$. Typical polymer chain concentration values are approximately in 0.1-2.0 $nm \cdot nm^3$ range, corresponding to a very accessible and a very dense gel phase, respectively.

In both models, however, the existence of cylindrical pores with different diameters or of gel domains with different polymer chain concentrations must be taken into account for a more realistic description of the system. On these grounds Jeřábek, [1] proposed Equation 4 to model the exclusion curve of the eluate whose (known) diameter is d:

$$V_e(d) = V_0 + \sum_{n=1}^{N} K_n(d, d_n) V_n \tag{4}$$

where n denotes a single pore fraction or gel domain with d_n as the characteristic dimension (the diameter for cylindrical pores, the local polymer chain concentration for the Ogston model). If a discrete number (N) of d_n are chosen and k solutes ($k \geq N$) are eluted, Equation 4 transforms into a system of N linear equation in V_n. As its analytical solution could give some negative values of V_n, which has no physical meaning numeric solution are preferred. They are obtained upon minimization of the sum of the squared differences between the left and right sides of Eq. 4, bounding the roots to null or positive values.

For sulfonated styrenic ion exchangers, ISEC measurements are performed in a 0.2M sodium sulfate solution (to suppress enthalpic interactions between standard solutes and polymer framework), by using D_2O, sugars (ribose, sucrose, xylose and raffinose) and dextranes (with molecular weight of 1500, 6000 and $6 \cdot 10^6$ in this investigation). The elution curves for these standard solutes for Gel_H_2SO_4, Gel_oleum and Gel_$HClSO_3$ are reported in Figure S1.

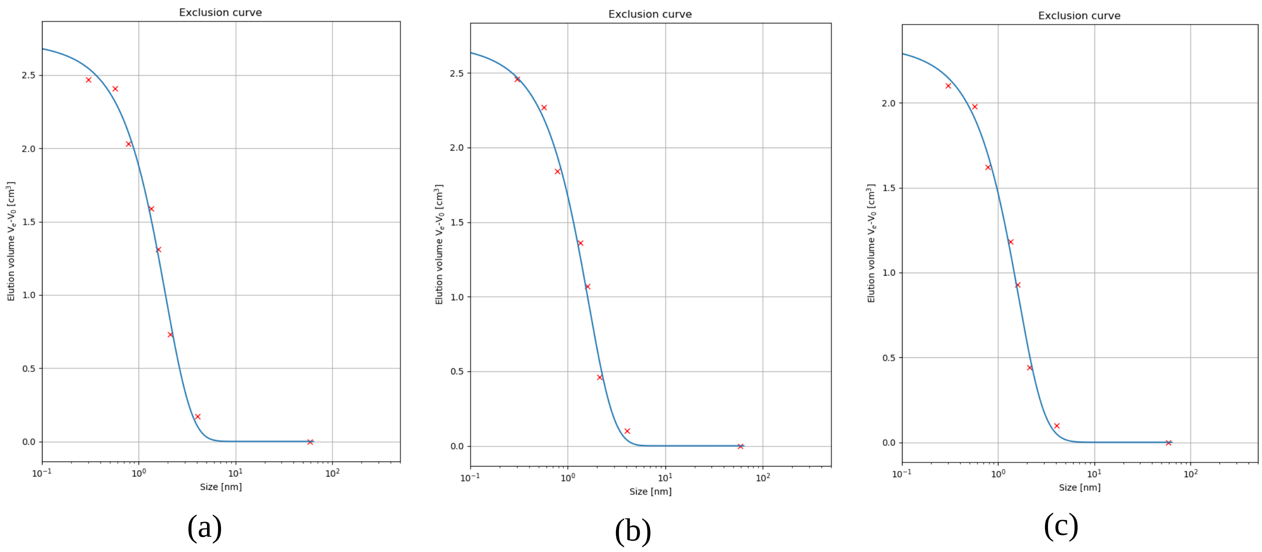

Figure S1. Elution curves for Gel_H_2SO_4 (a), Gel_oleum (b) and Gel_$HClSO_3$ (c).

References

1. Jeřábek, K. Determination of pore volume distribution from size exclusion chromatography data. *Anal. Chem.* **1985**, *57*, 1595–1597.
2. Jeřábek, K. Characterization of swollen polymer gels using size exclusion chromatography. *Anal. Chem.* **1985**, *57*, 1598–1602.
3. Jeřábek, K. Inverse Steric Exclusion Chromatography as a Tool for Morphology Characterization. In *Strategies in Size Exclusion Chromatography*; Potschka, M., Dubin, P.L., Eds.; American Chemical Society: Washington, DC, 1996; Vol. 635, pp. 211–224 ISBN 978-0-8412-3414-7.
4. Jeřábek, K.; Hanková, L.; Holub, L. Working-state morphologies of ion exchange catalysts and their influence on reaction kinetics. *J. Mol. Catal. Chem.* **2010**, *333*, 109–113.
5. Zecca, M.; Centomo, P.; Corain, B. CHAPTER 10 - Metal Nanoclusters Supported on Cross-Linked Functional Polymers: A Class of Emerging Metal Catalysts. In *Metal Nanoclusters in Catalysis and Materials Science*; Corain, B., Schmid, G., Toshima, N., Eds.; Elsevier: Amsterdam, 2008; pp. 201–232 ISBN 978-0-444-53057-8.
6. Corain, B.; Jeřábek, K.; Centomo, P.; Canton, P. Generation of Size-Controlled Pd0 Nanoclusters inside Nanoporous Domains of Gel-Type Resins: Diverse and Convergent Evidence That Supports a Strategy of Template-Controlled Synthesis. *Angew. Chem. Int. Ed.* **2004**, *43*, 959–962.
7. Ogston, A.G. The spaces in a uniform random suspension of fibres. *Trans. Faraday Soc.* **1958**, *54*, 1754–1757.
